# Health technology assessment in traditional and complementary medicine: a scoping review of international activity and examples of acupuncture

**DOI:** 10.1017/S0266462324000151

**Published:** 2024-04-05

**Authors:** Dan-Dan Ai, Bin-Yan Sui, Cheng-A-Xin Duan, Qian Xu, Kun Zhao

**Affiliations:** 1 Department of Health Technology Assessment, Beijing Health Economic Association, Beijing, China; 2 Department of Health Technology Assessment, China National Health Development Research Center, Beijing, China; 3 Vanke School of Public Health, Tsinghua University, Beijing, China; 4 Institute for Healthy China, Tsinghua University, Beijing, China

**Keywords:** traditional and complementary medicine, health technology assessment, acupuncture, value evaluation, scoping review

## Abstract

**Background:**

Traditional therapies are crucial in maintaining and improving human well-being. China’s healthcare policymakers are attempting to use health technology assessment (HTA) as a decision-making supportive tool. The value assessment framework for Chinese patent medicine (CPM) has been developed and is being adopted and validated widely by research institutions. Subsequently, the healthcare decision-makers particularly hanker for the value framework of traditional non-pharmacological therapies.

**Methods:**

To construct a practical value framework for traditional non-pharmacological therapies, a scoping review methodology was adopted to identify the evaluation domains and obstacles. A search, screening, and analysis process was conducted according to the Preferred Reporting Items for Systematic Reviews and Meta-Analyses extension for Scoping Reviews (PRISMA-ScR). Evidence was retrieved from scientific databases and HTA agencies’ websites.

**Results:**

The search strategy identified 5 guidelines records and 17 acupuncture HTA reports. By synthesizing the valuable reports of CPM and acupuncture evaluation in representative countries, this study found that Mainland China was promoting the comprehensive value assessment of CPM, whereas the United Kingdom, Singapore, Canada, the United States, and Malaysia had carried out the HTA evaluation of acupuncture for various conditions among which chronic pain was the most common. UK and Singapore applied the HTA results to support acupuncture reimbursement decisions. Three domains, including safety, effectiveness, and economy, were commonly adopted. The identified biggest challenge of evaluating traditional non-pharmacological therapies is the scarce high-quality clinical evidence.

**Conclusions:**

This study identified value domains and issues of traditional therapies, and pointed out future research implications, to promote the development value framework of traditional therapies.

## Introduction

Traditional, complementary, and integrative medicine (TCIM) has demonstrated its unique advantages and values in improving people’s health, and 170 of 194 (88 percent) WHO member states have admitted the formal use of TCIM within their health systems in an investigation (1;2). TCIM is an integrated conception, encompassing three parts: “traditional” therapy is viewed as a total sum of historical knowledge and beliefs unique to different ethnic groups, “complementary” therapy is defined as a non-mainstream practice used together with conventional medicine, while an “integrative” therapy is a combination of complementary approaches used in conjunction with conventional medicine (3). Among these, acupuncture has witnessed thriving and prosperous worldwide, though this technique was originally a feature of traditional Chinese medicine. According to the WHO’s survey including 129 countries, 80 percent of them recognized the use of acupuncture, with 18 countries reimbursing acupuncture in their healthcare service system (1).

To promote the high-quality development of TCIM, plenty of policies touching on clinical efficacy evaluation and value-based HTA have been introduced in China. Those evaluation systems are designed to showcase the theories, features, and value of TCIM, and eventually to inform the reimbursement decision-making and pricing of TCIM services (4). Furthermore, the value assessment framework for Chinese patent medicine has been issued by an academic institution. Whereas, there is no value framework for non-pharmacological therapies up to now. Therefore, Chinese healthcare decision-makers are pretty eager to use such tools to support their policy adjustment.

Apparently, to galvanize the reviving of TCIM, it is imperative to incorporate more traditional medicine services into health insurance coverage based on the high-quality value evaluation evidence (5). Consequently, It is necessary and valuable to conduct a scoping review of HTA practice in TCIM to have a general understanding of the evaluation dimensions and challenges. Given the wide adoption of acupuncture, the HTA reports of acupuncture can be more easily accessible than those of other traditional non-pharmacological therapies. It can bring more practical insights to develop the value assessment framework of traditional non-pharmacological technology by reviewing and synthesizing value domains and weaknesses mentioned in HTA reports of acupuncture treated for various diseases. We hope that the value framework can conduce to elucidate the potential value of traditional non-pharmacological therapies for disease prevention, treatment, and rehabilitation, and inform healthcare management and reimbursement policy-making in China.

## Methods

### Study Design and Search Strategy

To outline the evaluation framework of TCIM informing the value assessment and reimbursement decision making, a scoping review of the HTA guidelines for TCIM and HTA reports for acupuncture was conducted, complying with the methodological framework introduced by Arksey and O’Malley and refined by the Joanna Briggs Institute (6–8).

The PRISMA-ScR was adopted to elaborate this study and the PRISMA-ScR checklist can be found in Supplement 1. HTA, traditional medicine, complementary medicine, acupuncture, and other similar terms were used as keywords to construct the search strategy, retrieving the published literature on HTA in TCIM from relevant electronic databases, such as Medline (PUBMED), EMBASE, Web of Science, the International HTA database hosted by INAHTA, CNKI, Wanfang, and HTA agencies, including the National Institute for Health and Care Excellence (NICE), the Agency for Care Effectiveness (ACE), the Canadian Agency for Drugs and Technologies in Health (CADTH), the Institute for Clinical and Economic Review (ICER), the Scottish Medicines Consortium (SMC), the Swedish Agency for Health Technology Assessment and Assessment of Social Services (SBU) from inception to January 2023. The full search strategy can be found in Supplement 2. The scoping review protocol was not registered in a registry.

### Study Selection

Given the purpose was to pay attention to the HTA domains of TCIM and the HTA differences between TCIM and modern medical products, studies focusing on guidelines or standards specific to TCIM were included. In consideration of scarce assessment guidelines of TCIM, HTA reports of acupuncture for any diseases in the context of HTA organizations or agencies or other bodies worldwide were included as well, used to summarize HTA domains and characteristics of TCIM. HTA reports written in Chinese or English and available full texts were included. The detailed inclusion and exclusion criteria are shown in Table 1. The preliminary screening by titles and abstracts as well as the final screening by full texts was performed by two independent researchers (D.D. Ai and C.A.X. Duan) to determine the eventually included literature and potential disagreements were solved by a third author (B.Y. Sui).

**Table 1.** Inclusion and exclusion criteria

	Inclusion criteria	Exclusion criteria
Intervention	Guideline: specific to traditional, complementary, and integrative medicine Case study: Acupuncture or dry needling	General guidelines
Study type	Guidelines or consensuses or standards for TCIM HTA reports for acupuncture	Systematic reviews or Economic evaluations or for single traditional technology
Context	HTA agencies or bodies worldwide	–
Language	Chinese, English	Any other languages

### Data Extraction

A predefined qualitative data form encompassing report characteristics, HTA agency, country, year of publication, HTA domains, population, intervention and comparator, results, limitations, and implications were used to extract key information by two independent researchers.

### Data Synthesis

A descriptive analysis and a narrative synthesis were performed employing tables to synthesize the HTA domains and the main characteristics of each report.

## Results

### Reports Selection

**Figure 1.**
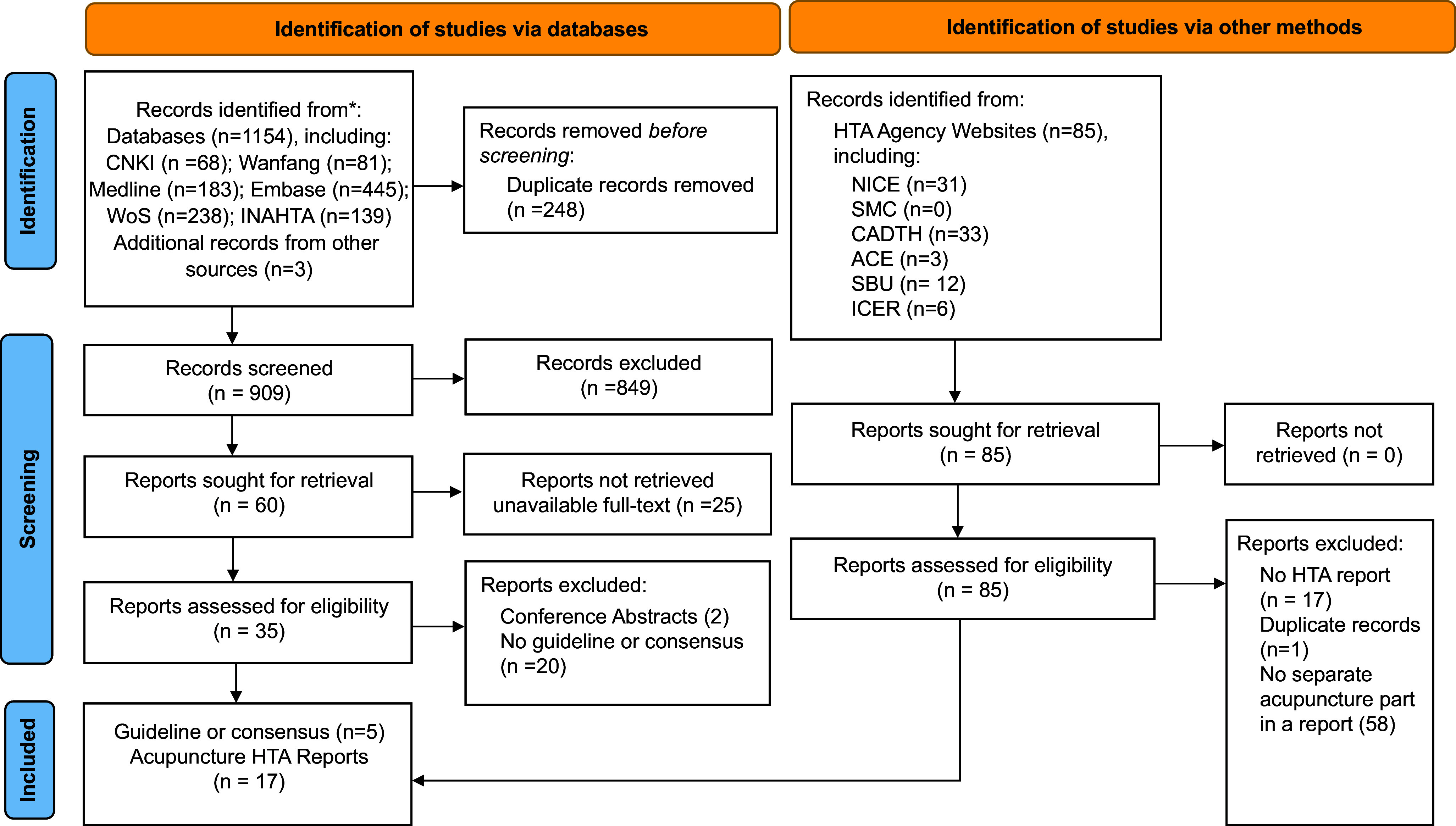
PRISMA flow diagram.

Overall, of the 993 records retrieved after deduplication, 971 were screened out since they were studies without acupuncture as an intervention, studies focusing on a narrative review of HTA in traditional medicine, or no guideline or consensus. Finally, 5 guideline or consensus records from China (9–13) and 17 acupuncture HTA reports from international HTA agencies (14–30) were included in the review. The full selection process is depicted in Figure 1.

### Description of Included Studies

#### Outline of HTA Guidelines or Consensus in TCIM

Five guidelines or consensus records (9–13) about value assessment of Chinese patent medicine released by Chinese academic groups were identified, while there were no published HTA guidelines or consensus for TCIM in other countries and regions. In 2021, the China National Health Commission (NHC) issued the Management Guideline for the Clinical Comprehensive Evaluation of Drugs, which involved six domains, including safety, effectiveness, economy, innovation, suitability, accessibility, to measure the value of a drug based on the HTA methods and multiple-criteria decision analysis (MCDA) to improve clinical rational use of medicines (31). Since then, China healthcare sectors and academic groups have been promoting clinical comprehensive evaluation of drugs. With that management guideline, in the field of TCIM, the Chinese Association of Traditional Chinese Medicine, the Chinese Academy of Chinese Medical Sciences and other institutions drafted or promulgated technical evaluation guidelines or technical specifications, and carried out clinical comprehensive evaluation of specific Chinese patent medicines, of which the evaluation dimensions were basically consistent with management guidelines issued by NHC (9–13). Notably, some institutions added up Chinese medicine-specific indicators, such as the traditional Chinese medicine theory, human use experiences, and so on (11;12). In another technical guideline developed by the Institute of Clinical Basic Medical Sciences of the Chinese Academy of Chinese Medical Sciences, the three dimensions, namely safety, effectiveness, and economy, were drawn up from the national clinical comprehensive evaluation, and the other dimensions (application, science and standards) reflecting the features of TCM were proposed by Delphi method (see Table 2) (10;13).

**Table 2.** Information table for comprehensive evaluation of Chinese patent medicine

References	Safety	Effectiveness	Economy	Innovation	Suitability	Accessibility	Specialties of TCM	Application	Science	Standards
Yuan (9)	√	√	√	√	√	√				
Zhang (11;12)	√	√	√	√	√	√	√			
Zhang (10;13)	√	√	√					√	√	√

Internationally, even though some HTA of TCIM were conducted to provide evidence for medical insurance coverage decision-making, there was currently no published value assessment framework or economic evaluation method aimed at traditional medicine or non-pharmacological therapies in other countries and regions. South Korea, Switzerland, Singapore, and the United Kingdom have attempted to apply HTA for traditional and complemental medicine to inform medical insurance reimbursement. In Korea, *Hanbang* services have been covered in the benefits schedule in the country’s National Health Insurance (NHI) since 1987. Currently, health insurance benefits of *Hanbang* are especially focused on treatments such as acupuncture, moxibustion, and cupping, while herbal medicines coverage is relatively rare. To promote *Hanbang*, it is necessary to prioritize the extension of health benefits to herbal medicines and traditional techniques with a lower rate of coverage. Consequently, the National Development Institute of Korean Medicine (NIKOM) has developed a strategic plan that includes the development of economic valuation guidelines specific to *Hanbang* services under the current HTA system. In line with the evidence-based decision making and value purchase principles, the government led the formulation of *Hanbang* clinical practice guidelines for services, the first batch of which contained 27 diseases (5). In Switzerland, reimbursement for traditional and complemental medicine after 2017 will depend on the results of the evaluation projects and international HTAs (2). A systematic review of technical assessments of traditional medicine conducted by HiTAP, which included peer-reviewed articles, grey literature, and regional or international guidelines on the evaluation of herbal products and traditional and complementary medical practices found there was no organized framework to adapt economic evaluation methods to the unique features of TCIM. The reporting of costs and health-related quality of life remained uncommon in TCIM studies. There was a call for updating the guidelines on the evaluation of TCIM and developing better evaluation frameworks (32).

#### Examples of HTA in Acupuncture

The scoping review identified 17 published acupuncture HTA reports from five countries, including the United Kingdom (8) (22–29), the United States (3) (15;16), (21), Canada (3) (17;18;30), Singapore (1) (14), Malaysia (1) (20), and one international academic organization, Cochrane (1) (19), between 2003 and 2022. The involved conditions were mainly chronic pain, and otherwise included allergic rhinitis, induction, irritable bowel syndrome, and stable angina. Usual care or a sham/placebo acupuncture were the most common comparators. Detailed characteristics of all included reports are provided in Table 3.

**Table 3.** Summary characteristics of included acupuncture HTA reports

Country	Year	HTA organization	Population	Intervention	Comparator	Outcome main results	Challenges and issues
UK (25)	2021	NICE	Chronic primary pain	Acupuncture/dry needling Electro acupuncture	Placebo/sham Usual care	The recommendation was caveated with information on the number of hours of staff time, and banding of staff that would make the delivery of acupuncture cost–effective	Low to very low evidence quality. Lack of blinding Mostly subjective outcomes resulted in a high risk of performance bias. Uncertainties about the cost–effectiveness of acupuncture, as there was a limited evidence base and there were uncertainties around the cost of the intervention
UK (28)	2022	NICE	Osteoarthritis	Acupuncture/dry needling Electro–acupuncture	Sham No intervention	The adverse events data for these trials was limited as this was generally found in small studies with a short follow–up time	High to very low evidence quality due to inconsistency and imprecision. Inconsistent types of sham acupuncture between studies, increasing the uncertainty of the results Complex nature of acupuncture treatment: the effect of the relationship between the health care professional and the person with osteoarthritis
UK (27)	2021	NICE	Primary headaches	Acupuncture	Sham	Some evidence for improvements in headache days and responder rate Treatment reactions after acupuncture needling were common. The risk of serious side effects was low Acupuncture was cost–effective when compared to no treatment in people with migraine or tension–type headache (£12,381/QALY) from a published literature based on an RCT An original cost–effectiveness analysis showed that acupuncture was more cost–effective than no treatment when 10 or fewer sessions were provided	Low and very low–quality evidence Economic evidence had minor limitations and partial applicability
UK (24)	2017	NICE	Irritable bowel syndrome (IBS)	Single acupuncture Combination acupuncture	Sham/placebo Other type of treatment	No significant effect of acupuncture on IBS global symptoms, pain, and quality of life compared with placebo	Limited evidence of potentially serious adverse effects associated with acupuncture treatments
UK (26)	2021	NICE	Women undergoing induction	Acupuncture	Usual care	Evidence was insufficient to determine the effectiveness of acupuncture in cervical priming/induction of labor	Insufficient evidence
UK (23)	2016	NICE	Stable angina	Acupuncture	Sham	The evidence was mixed: One RCT showed some improvement in angina and exercise test variables when compared to tablet placebo. However, there was no improvement in angina or exercise test variables in two RCTs that compared acupuncture to sham	Small sample size (<50 patients) Without long–term follow–up outcomes The methodology of the trials was not well reported and the derived data was not analyzable
UK (22)	2017	NIHR	Osteoarthritis Headaches Shoulder pain Back or neck pain	Acupuncture	Sham non–acupuncture	Acupuncture was more effective than sham acupuncture, and was better than standard medical care for all of these chronic pain conditions when based on high–quality trial evidence Acupuncture was also cost–effective if only high–quality trials were analyzed. When synthesizing all trials, including both low– and high–quality trials, transcutaneous electrical nerve stimulation was cost–effective	Limited data regarding the long–term effects of many non–pharmacological interventions
Singapore (14)	2020	ACE	Low back pain and neck pain	Acupuncture±standard care	Standard care Sham No treatment	The effectiveness of acupuncture was unclear for chronic arthritis, osteoarthritis, shoulder–arm syndrome, and epicondylitis when compared with alternative treatments The overall applicability of economic evaluation estimates to the local context was unclear	Poor quality of evidence Small sample size Unclear clinically relevant benefits Considerable heterogeneity among studies Poor reporting of acupuncture treatment details
Canada (18)	2019	CADTH	Chronic non–cancer pain	Acupuncture	Pharmacological interventions No treatment Usual care	The clinical effectiveness results and recommendations were variable depending on the patient population. Acupuncture decreased pain intensity in chronic prostatitis/chronic pelvic pain syndrome, chronic headache, chronic neck pain, chronic shoulder pain, sciatica, myofascial pain, hip osteoarthritis, knee osteoarthritis, and osteoarthritis when compared to sham. For other pain conditions, the evidence regarding the clinical effectiveness was mixed. No firm conclusions can be made regarding the relative cost–effectiveness	Few high–quality primary studies and many low–quality primary studies High risk of bias: non–randomized or unconcealed allocation, lack of blinding or maintenance, heterogeneous population or treatment, non–standardized additional treatments, or even insufficient reporting of risk of bias items Outcomes lack of standardization, clinical validation, length of appropriate follow–up, and minimum clinically important difference Economic studies from outside of Canada might be less applicable within Canada
Canada (17)	2016	CADTH	Musculoskeletal pain Joint disorders Joint pain Derangement of joints Chronic tendinosis Tendinopathy	Dry needling±injection	No comparator Usual care	Insufficient evidence on the effects of dry needling on other outcomes, such as range of movement or quality of life	Most methodologically flawed primary studies: inadequate blinding, underpowered to accurately indicate treatment effects, heterogeneous patient populations, various outcome measures and length of follow–up, and non–standardized treatment and comparator interventions MCID was not defined, and the results focused only on statistically significant changes
US (21)	2017	ICER	Chronic low back or neck pain	Cognitive and mind–body therapies, including: Acupuncture CBT MBSR Yoga Tai chi	Usual care Sham/placebo	Small to moderate improvements in function and pain compared with usual care immediately following the completion of therapy The differences in outcomes were smaller and often non–significant clinically when compared to sham acupuncture, suggesting that much of the benefit may be from the placebo effect The incremental cost of achieving one case of improved pain over the 5–year time horizon relative to usual care ranged from $660 for yoga to approximately $15,800 for CBT (<$100,000/QALY)	Difficult to interpret the clinical significance of average changes in continuous measures of function, quality of life, and pain. Did not model varying treatment effectiveness over time due to the deficit of long–term trial data, did not model subsequent lines of intervention for individuals who experienced a recurrence of low back pain due to a lack of published evidence on this estimate
Malaysia (20)	2020	MaHTAS	Neurological disorders (including headache, refractory neuralgia, Bell’s palsy, post–stroke, Guillain Barre and transverse myelitis)	Acupuncture Traditional body needling Moxibustion Electro–acupuncture Laser acupuncture	Sham/placebo Scalp acupuncture Other conventional treatment	None of the clinical trials reported any serious adverse events. Acupuncture was suggested to be clinically relevant benefit and cost–effective in certain sectors	Poor quality of the included trials: high risk of bias due to inappropriate randomized sequence generation, lack of allocation concealment, inadequate level of blinding, and poor description of patient withdrawals from the studies
Canada (30)	2022	WorkSafeBC	Lateral Elbow Pain	Dry Needling	Sham Other active treatments No comparator	No high–quality evidence supporting the efficacy and/or effectiveness of dry needling	Most studies with very low to low study quality: methodological limitations and imprecision, low numbers of participants, and inadequate blinding
International organization (19)	2018	Cochrane	Carpal tunnel syndrome	Acupuncture Acupuncture–related interventions	Sham Other active treatments	Acupuncture was associated with no serious adverse events, or reported discomfort, pain, local anesthesia, and temporary skin bruises	Low or very low evidence Most evidence was short–term
UK (29)	2007	WMHTAC	Allergic rhinitis	Acupuncture	Sham±standard care	Insufficient evidence on the clinical effectiveness to support or refute its use in patients with allergic rhinitis Acupuncture was not associated with any additional adverse events	Poor quality and the inconclusive results
US (15)	2003	AHRQ	Fibromyalgia	Acupuncture Electro–acupuncture	Sham	Insufficient evidence to conclude that acupuncture had efficacy for the treatment of fibromyalgia	Small sample size Inappropriateness of outcome measures Complexities of acupuncture: different types of acupuncture, different systems for choosing sites, and variability in the technique of needle insertion and manipulation
US (16)	2003	AHRQ	Osteoarthritis	Acupuncture Electro–acupuncture	Sham Other active treatments	–Most studies did not find a benefit for acupuncture compared to sham acupuncture	Small sample size Underpowered to measure statistically significant differences between the interventions Lack of blinding Lack of description of the handling of dropouts and withdrawals No formal test statistics

ACE, Agency for Care Effectiveness; AHRQ, Agency for Healthcare Research and Quality; CADTH, Canadian Agency for Drugs and Technologies in Health; ICER, Institute for Clinical and Economic Review; MaHTAS, Malaysian Health Technology Assessment Section; NICE, National Institute for Health and Care Excellence; NIHR, National Institute for Health Research; WMHTAC, West Midlands Health Technology Assessment Collaboration.

The evaluation dimensions mainly contained safety, effectiveness, cost-effectiveness, medical resources consumed, and guideline recommendations (see Table 4). Agency for Care Effectiveness (ACE), a Singaporean HTA organization, also took three other factors, the clinical needs and disease characteristics, organizational feasibility, and ethical or social issues, into consideration (14). The assessment report of the Institute for Clinical and Economic Review (ICER) in US mentions health insurance coverage, other important benefits or risks (21). Generally speaking, all evaluation bodies attached importance to the quality of the evidence of safety and effectiveness. In Singapore, guided by the medical device technology guidelines, ACE conducted the evaluation of acupuncture, which predicted the resources utilization with the technology’s annual cost and the number of patients who may benefit from it. The organizational feasibility assessment aimed to identify barriers and facilitate technology’s adaptation into their public healthcare system, and any organizational factors that might influence the technology’s performance or use in clinical practice.

**Table 4.** HTA domains of acupuncture evaluation

HTA organization	National health system	Safety	Efficacy/effectiveness	Cost-effectiveness	Consumed medical resources	Guidelines recommendations	Clinical needs and disease features	Organizational feasibility	Health insurance coverage
ACE (10)	Public	√	√	√	√	–	√	√	–
NICE (19–24)	Public	√	√	√	√	√	–	–	–
CADTH (13;14)	Public	√	√	√	–	√	–	–	–
ICER (17)	Private	√	√	√	√	√	–	–	√
MaHTAS (16)	Public	√	√	√	–	–	–	–	–

The clinical comparative effectiveness results varied from different patient population in the comparison of acupuncture with placebo or sham acupuncture (14–30). When only synthesizing high-quality clinical evidence, acupuncture was more effective than sham acupuncture or usual care for all of these chronic pain conditions. However, there were plenty of low-quality clinical studies, resulting the inconclusive conclusions. Methodological flaws leading to the generally downgraded evidence quality, were pointed out most frequently. The evidence quality was typically downgraded due to risk of bias and imprecision, like small sample size, inappropriate randomized sequence generation, lack of allocation concealment, inadequate level of blinding, various types of sham acupuncture, considerable heterogeneity among studies, poor reporting of treatment details about interventions and comparators, high placebo response, and non-standardized additional treatments, which finally resulted in unclear and inconsistent clinically relevant benefits. Plus, subjective measures lack of standardization, clinical validation, length of appropriate follow-up, and minimum clinically important difference (MCID) were also challenged. Typically, the continuous outcome results focused only on statistically significant changes, so it was difficult to interpret the clinical significance of average changes in measures of function, quality of life, and pain. Furthermore, although most of the therapies were for chronic pain, long-term follow-up clinical outcomes were not reported in these studies.

Otherwise, the adverse events data for these trials was limited as this was generally found in a few studies with a short follow-up time and so it was unclear whether this was representative of the events expected to be seen in real-life clinical practice. Also, severe adverse reactions were scarce. Considering the beforementioned limitations about clinical effectiveness, there was a limited evidence, and thus many extrapolation assumptions applied in the cost-effectiveness analysis models were lack of robust evidence. The cost-effectiveness results of acupuncture compared with no treatment were inconsistent among various studies, and by limiting the treatment session and duration, acupuncture was likely to become a cost-effective intervention within the country’s threshold (25;27).

## Discussion

### Summary of Main Findings

Overall, there were still no other HTA guideline or consensus of TCIM besides clinical comprehensive evaluation guideline published by academic agencies in China. Typically, the evaluation of TCIM adhered to general guidelines drafted by HTA agencies. The evaluation domains included safety, effectiveness, economy (involving cost effectiveness and consumed medical resources), innovation, suitability, accessibility, specialties of TCM, application, science, standards, guidelines recommendations, clinical needs and disease features, organizational feasibility, health insurance coverage, among which safety, effectiveness, and economy were the most frequent domains.

The role and function of TCIM HTA in policy-making varied across the countries. Despite the fact that forty-five of the WHO member states currently had health insurance coverage for traditional medicine and T&CM practices (e.g., acupuncture and chiropractic) (2). Few countries or regions applied HTA to support medical insurance decision-making for traditional medical services (33), and countries with well-developed HTA knowledge translation mechanism such as the United Kingdom, Singapore, and Switzerland are exploring the use of HTA to support medical insurance access for acupuncture (2;14;22–29). With the need to cover more tradition medical technologies in insurance coverage, evidence-based assessment of its value for money would become a matter of growing importance. There was an appeal to focus on developing better frameworks for evaluating TCIM in order to obtain regulatory approval and/or reimbursement (32).

Compared with other narrative reviews about the status and challenges of HTA and economic evaluation in traditional Chinese medicine and acupuncture, this study qualitatively synthesized the value evaluation domains and issues by systematic retrieving evaluation guidelines of TCIM and HTA reports of acupuncture according to the methodology framework of scoping review. It was the first time to synthesize the value evaluation domains of TCIM, even though those domains were mostly consistent with general drug or medical device technology evaluation domains. However, some researchers were appealing and making efforts to add traditional medical-related factors to value framework (10–13;32). Currently, it is evident that economic evaluation plays a limited role in reimbursement decisions for acupuncture (34). Given that specific healthcare system, costing, healthcare service pathway, and population health preferences, it was difficult to generalize the economic evaluation results to other countries. In terms of the issues, apart from the study design, lack of long-term effectiveness, and poor reporting quality, previous studies also depicted other challenges, such as cost measurement and PICO definition. It was difficult to accurately collect due to dosage and duration adjustment based on the symptoms and characteristics of the patient at each visit, self-pricing and unpublished price. And “Intervention” was not easy to be determined because the number and dosage of traditional medical service were adjusted based on patients’ characteristics and doctors’ experience (33;35).

### Limitations

There were also several limitations in this article. First of all, studies might have been missed because only peer-reviewed publications or HTA reports released by HTA agencies and written in English or Chinese were considered. Therefore, the included literatures might not be completely representative of the research available. In addition, the study limitations were mainly due to the applicability of generalizing acupuncture assessment domains to traditional non-pharmacological technology assessment, especially considering the specialities of other Chinese medical technology, like tuina, acupotomy, and so on. Furthermore, some included HTA reports might contain inadequate information, leading to insufficient data extraction of the study characteristics of interest, which also restricted the extrapolation of results.

### Future Research and Implication

It can be expected value framework of TCIM could expedite the generation and accumulation of high-quality primary evidence. As human-use experience plays an extremely important role in the development of TCIM, real-world diagnosis and treatment data have significant advantages in the effectiveness evaluation of TCIM, which also conduce to address the questions about blindness in RCT and complex peculiarity of traditional non-pharmacological technology to enhance the quality of clinical effectiveness evidence. The well-designed pragmatic randomized clinical trials and other observational studies are very suitable to consider the features of TCIM, which can not only standardize, but also reflect the characteristics of dialectical treatment of Chinese medicine. Recently, eRCT-pRCT-RCDOS-REGOS,^1^ a stepwise progressive clinical effectiveness evaluation system of TCIM was proposed, aimed to enhance study quality by broadening the study populations and interventions to assess the net benefits in more contexts, such as practitioner proficiency, equipment quality, and others (36). For continuous outcomes, particularly when using a patient-reported outcome measurement tool, researchers should report both statistical and clinical significance, taking MCID into consideration. Considering the wide usage of subject clinical outcomes in the clinical value assessment of TCIM, the study regarding MCID of clinical outcome measures should be encouraged. Categorical measures reporting the proportion of patients achieving a clinically meaningful improvement in function, quality of life, and pain are more useful and should be reported in addition to average group changes. Those factors are beneficial for generating high-quality value evidence for TCIM.

## Conclusion

This scoping review provided an overview of the HTA guideline of TCIM and acupuncture HTA reports across various countries. With China vigorously promoting the inheritance and innovation of traditional Chinese medicine and increasingly emphasizing evidence-based decision making, the demand for HTA of TCIM is on the rise, providing evidence for the price adjustment of traditional medical services and the coverage decisions of Chinese patent medicine. Currently, domestic studies about comprehensive value assessment of Chinese patent medicine are on the rise, while few studies address the question of how to assess the value of traditional non-pharmacological technology in order to pursue the decision-makers to purchase traditional and complementary medical services widely. International HTA agencies have published several evaluation reports of acupuncture for various conditions, and the results were used to inform medical insurance reimbursement decisions. The most common assessment domains are clinical effectiveness, safety, and economy. In the process of evidence synthesis, however, some issues, such as poor clinical evidence quality, sophisticated costing, lack of long-term effectiveness, and so on, set obstacles to conclude robust value evaluation results for traditional non-pharmacological technology. In the future, more efforts should be put on how to transform tons of human-usage experience in real-world clinical practice into high-quality evidence. It is a valuable topic to determine if economic evaluation best practice demonstration case is needed for traditional non-pharmacological technology considering these unique features like short-term intervention and potential long-term benefits for physical and mental health, in order to improve study quality and inform scientific decision making.

## Supporting information

Ai et al. supplementary material 1 Ai et al. supplementary material

Ai et al. supplementary material 2 Ai et al. supplementary material

## Footnotes

1 eRCT, explanatory randomized controlled trial; pRCT, pragmatic randomized controlled trial; RCDOS, observational studies using routinely collected data; REGOS, observational studies using patient registry data.

## Supplementary material

The supplementary material for this article can be found at http://doi.org/10.1017/S0266462324000151.

## References

[1] World Health Organization. WHO traditional medicine strategy: 2014–2023. 2013. Available from: https://www.who.int/publications/i/item/9789241506096 (accessed Jan 19, 2024).

[2] World Health Organization. WHO global report on traditional and complementary medicine 2019. Available from https://apps.who.int/iris/handle/10665/312342 (accessed Mar 31, 2023).

[3] Ng JY, Hilal A, Maini I. What traditional, complementary, and integrative medicine recommendations exist across osteoporosis clinical practice guidelines? A systematic review and quality assessment. Integr Med Res. 2022;11(2):100803. 34840950 10.1016/j.imr.2021.100803 PMC8605333

[4] General Office of the State Council of the People’s Republic of China. A new five-year plan for the development of traditional Chinese medicine. Available from: http://www.gov.cn/zhengce/content/2022-03/29/content_5682255.htm (accessed Mar 31, 2023).

[5] Kwon H-Y, Kim H-L, Kim J. Application of the health technology assessment in Korean traditional medicines. J Altern Complement Med. 2021;27(1):58–65. 33136429 10.1089/acm.2020.0359

[6] Peters MD, Godfrey CM, Khalil H, McInerney P, Parker D, Soares CB. Guidance for conducting systematic scoping reviews. JBI Evid Implement. 2015;13(3):141–146. 10.1097/XEB.0000000000000050 26134548

[7] Aromataris EMZ. JBI manual for evidence synthesis: JBI. 2020. 10.46658/JBIMES-20-01.

[8] Arksey H, O’Malley L. Scoping studies: Towards a methodological framework. Int J Soc Res Methodol. 2005;8(1):19–32.

[9] Yuan WA, Zhang JH, Liu JP, et al. Guideline for clinical comprehensive evaluation of Chinese patent medicine (2022 version). Chin J Chinese Mat Med. 2023;48(01):256–264. 10.19540/j.cnki.cjcmm.20220922.501 36725278

[10] Zhang HL, Liang N, Chen YX, et al. Interpretation of the guideline for a multi-dimensional and multi-criteria comprehensive evaluation for Chinese patent medicine. Chin J Evid Based Med. 2022;22(07):762–767.

[11] Zhang Q, Wang ZF, Xie YM, et al. Technical specification for clinical comprehensive evaluation of Chinese patent medicine. World Chinese Med. 2021;16(22):3394–3397.

[12] Zhang Q, Wang ZF, Xie YM, et al. Report standards for clinical comprehensive evaluation of Chinese patent medicine. China J Chinese Mat Med. 2021;46(23):6062–6067. 10.19540/j.cnki.cjcmm.20210930.502 34951233

[13] Institute of Basic Research in Clinical Medicine, China Academy of Chinese Medical Sciences, Dongzhimen Hospital, Beijing University of Chinese Medicine, Guidelines and Standards Research Center of Chinese Medical Association Publishing House, China Information Association for Traditional Chinese Medicine and Pharmacy Clinical Research Information Association, China Association for Standardization, Branch of Chinese Medicine. Guideline for multi-dimensional and multi-criteria comprehensive evaluation of Chinese patent medicine. Chin J Evid Based Med. 2022;22(07):751–755.

[14] Agency for Care Effectiveness. Acupuncture for adults with low back pain and neck pain. Available from: https://www.ace-hta.gov.sg/healthcare-professionals/ace-technology-guidances/details/acupuncture-for-adults-with-low-back-pain-and-neck-pain (accessed Mar 31, 2023).

[15] Agency for Healthcare Research and Quality. Acupuncture for fibromyalgia. United States: Agency for Healthcare Research and Quality (AHRQ). 2003. Available from: https://www.cms.gov/medicare-coverage-database/view/technology-assessments.aspx?TAId=18 (accessed Mar 31, 2023).

[16] Agency for Healthcare Research and Quality. Acupuncture for osteoarthritis. United States: Agency for Healthcare Research and Quality (AHRQ). 2003. Available from: https://www.cms.gov/medicare-coverage-database/view/technology-assessments.aspx?TAId=19 (accessed Mar 31, 2023).

[17] Canadian Agency for Drugs and Technologies in Health. Dry needling and injection for musculoskeletal and joint disorders: A review of the clinical effectiveness, cost-effectiveness, and guidelines. Available from https://www.ncbi.nlm.nih.gov/books/NBK395711/ (accessed Mar 31, 2023). 27831670

[18] Canadian Agency for Drugs and Technologies in Health. Acupuncture for chronic non-cancer pain: A review of clinical effectiveness, cost effectiveness and guidelines. Available from: https://www.cadth.ca/sites/default/files/pdf/htis/2019/RC1202%20Acupuncture%20for%20Pain%20Final.pdf (accessed Mar 31, 2023). 31877002

[19] Choi GH, Wieland LS, Lee H, et al. Acupuncture and related interventions for the treatment of symptoms associated with carpal tunnel syndrome. Cochrane Database Syst Rev. 2018;12(12):Cd011215. 30521680 10.1002/14651858.CD011215.pub2 PMC6361189

[20] Fatin NM, Izzuna MMG. Acupuncture for headache, refractory neuralgia, Bell’s palsy, post-stroke, Guillain barre and transverse myelitis. Malaysia: Malaysian Health Technology Assessment (MaHTAS). 2020. Available from: https://www.moh.gov.my/index.php/database_stores/store_view_page/30/358 (accessed Mar 31, 2023).

[21] Institute for Clinical and Economic Review. Institute for clinical and economic review: Cognitive and mind-body therapies for chronic low back and neck pain: Effectiveness and value. Available from: https://icer.org/wp-content/uploads/2020/10/CTAF_LBNP_Final_Evidence_Report_110617.pdf (accessed Mar 31, 2023).

[22] MacPherson H, Vickers A, Bland M, et al. Acupuncture for chronic pain and depression in primary care: A programme of research. Programme Grants Appl Res. 2017;5(3):1–342. 28121095

[23] National Institute for Health and Care Excellence. Stable Angina. Available from: https://www.nice.org.uk/guidance/cg126/evidence/full-guideline-pdf-183176605 (accessed Mar 31, 2023).

[24] National Institute for Health and Care Excellence. Irritable bowel syndrome in adults: Diagnosis and management of irritable bowel syndrome in primary care. Available from: https://www.nice.org.uk/guidance/cg61/evidence/full-guidance-pdf-196701661 (accessed Mar 31, 2023).

[25] National Institute for Health and Care Excellence. Chronic pain (primary and secondary) in over 16s: assessment of all chronic pain and management of chronic primary pain. Available from: https://www.nice.org.uk/guidance/cg61/evidence/full-guidance-pdf-196701661 (accessed Mar 31, 2023). 33939353

[26] National Institute for Health and Care Excellence. Induction of labour. Available from: https://www.nice.org.uk/guidance/ng207/evidence/full-guideline-july-2008-pdf-9266823757 (accessed Mar 31, 2023).

[27] National Institute for Health and Care Excellence. Headaches: Diagnosis and management of headaches in young people and adults. Available from: https://www.nice.org.uk/guidance/cg150/evidence/full-guideline-pdf-188258224 (accessed Mar 31, 2023).

[28] National Institute for Health and Care Excellence. Osteoarthritis in over 16s: Diagnosis and management. Available from: https://www.nice.org.uk/guidance/ng226/evidence/f-clinical-and-cost-effectiveness-of-acupuncture-for-people-with-osteoarthritis-pdf-11250452851 (accessed Mar 31, 2023). 36745715

[29] Roberts J. Acupuncture for allergic rhinitis 2007. Available from https://www.birmingham.ac.uk/research/activity/mds/projects/HaPS/PHEB/WMHTAC/REP/reports-list.aspx (accessed Mar 31, 2023).

[30] WorkSafeBC Evidence-Based Practice Group, Martin CW. Dry needling for lateral elbow pain. Canada: WorkSafeBC. 2022. Available from: https://www.worksafebc.com/en/resources/health-care-providers/guides/dry-needling-lateral-elbow-pain?lang=en (accessed Mar 31, 2023).

[31] China National Health Commission. Management guideline for the clinical comprehensive evaluation of drugs. Available from: http://www.nhc.gov.cn/yaozs/s2908/202107/532e20800a47415d84adf3797b0f4869.shtml (accessed Mar 31, 2023).

[32] Lin LW, Ananthakrishnan A, Teerawattananon Y. Evaluating traditional and complementary medicines: Where do we go from here. Int J Technol Assess Health Care. 2021;37(1):e45. 33729111 10.1017/S0266462321000179

[33] Chen Y. Health technology assessment and economic evaluation: Is it applicable for the traditional medicine. Int Med Res. 2022;11(1):100756. 10.1016/j.imr.2021.100756 PMC8358415 34401322

[34] Li H, Jin X, Herman PM, et al. Using economic evaluations to support acupuncture reimbursement decisions: Current evidence and gaps. BMJ. 2022;376:e067477. 35217521 10.1136/bmj-2021-067477 PMC8868047

[35] Yang Y, Tian K, Bai G, et al. Health technology assessment in traditional Chinese medicine in China: Current status, opportunities, and challenges. Global Health J. 2019;3(4):89–93.

[36] Sun X, Li L, Liu Y, et al. Assessing clinical effects of traditional Chinese medicine interventions: Moving beyond randomized controlled trials. Front Pharmacol. 2021;12:713071. 34557094 10.3389/fphar.2021.713071 PMC8452912

