# Supplementary material for: Health technology assessment in traditional and complementary medicine: a scoping review of international activity and examples of acupuncture
Source: Int J Technol Assess Health Care. 2024 Apr 5;40(1):e24. doi: 10.1017/S0266462324000151 (PMC11570047; doi:10.1017/S0266462324000151)
Supplement: Ai et al. supplementary material 2 — Ai et al. supplementary material [file S0266462324000151sup002.docx]

### Supplement 2 Search Strategy

##### Medline/Pubmed

| Search query | Results |
| --- | --- |
| ((complementary therapies[MeSH Terms]) OR (((((((((acupuncture[Title/Abstract]) OR (needling[Title/Abstract])) OR (Intramuscular stimulation therapy[Title/Abstract])) OR (moxibustion[Title/Abstract])) OR (chiropractic[Title/Abstract])) OR (moxa cautery[Title/Abstract])) OR (massage[Title/Abstract])) OR (cupping[Title/Abstract])) OR (((((((((((((Complementary therapies[Title/Abstract]) OR (Traditional Medicine[Title/Abstract])) OR (Complementary Medicine[Title/Abstract])) OR (Integrative Medicine[Title/Abstract])) OR (Traditional Chinese Medicine[Title/Abstract])) OR (TCM[Title/Abstract])) OR (Herbal Medicine[Title/Abstract])) OR (Traditional medicine technology[Title/Abstract])) OR (Herbal therap*[Title/Abstract])) OR (phytotherapy[Title/Abstract])) OR (Kampo[Title/Abstract])) OR (Ayurvedic formulations[Title/Abstract])) OR (naturopathic medicine[Title/Abstract])))) AND (("Technology Assessment, Biomedical"[Mesh]) OR (((((health technology assessment[Title/Abstract]) OR (HTA[Title/Abstract])) OR (rapid review[Title/Abstract])) OR (value framework*[Title/Abstract])) OR (Value Assessment Framework*[Title/Abstract]))) AND (chinese[Filter] OR english[Filter]) | 183 |

##### Web of Science

| # | Query | Results |
| --- | --- | --- |
| 1 | (((((((TS=(Complementary therapies)) OR TS=(Traditional, Complementary, and Integrative Medicine)) OR TS=(Traditional Chinese Medicine)) OR TS=(Traditional medicine technology)) OR TS=(Kampo)) OR TS=(acupuncture)) OR TS=(needling)) OR TS=(Intramuscular stimulation therapy) | 1277648 |
| 2 | ((((TS=("health technology assessment")) OR TS=(HTA)) OR TS=("rapid review")) OR TS=("value framework*")) OR TS=("value assessment framework*") | 17467 |
| 3 | #1 AND #2 | 263 |
| 4 | #1 AND #2 and English or Chinese | 238 |

##### Embase

| No. | Query | Results |
| --- | --- | --- |
| #20 | #18 AND ('article'/it OR 'review'/it) AND ([chinese]/lim OR [english]/lim) | 445 |
| #19 | #18 AND ('Article'/it OR 'Review'/it) | 463 |
| #18 | #16 AND #17 | 574 |
| #17 | #1 OR #3 OR #4 OR #5 OR #6 OR #7 OR #8 OR #9 | 441864 |
| #16 | #10 OR #11 OR #12 OR #13 OR #14 | 30490 |
| #14 | 'value assessment framework*' | 132 |
| #13 | 'value framework*' | 529 |
| #12 | 'rapid review'/exp OR 'rapid review' | 2068 |
| #11 | 'health technology assessment'/exp OR 'health technology assessment' | 28008 |
| #10 | 'biomedical technology assessment' | 16901 |
| #9 | 'dry needling'/exp OR 'dry needling' | 1234 |
| #8 | 'acupuncture'/exp OR 'acupuncture' | 66251 |
| #7 | 'kampo medicine (drug)'/exp OR 'kampo medicine (drug)' | 5000 |
| #6 | traditional AND ('medicine'/exp OR medicine) AND ('technology'/exp OR technology) | 60899 |
| #5 | 'traditional medicine'/exp OR 'traditional medicine' | 149384 |
| #4 | 'chinese medicine' | 207057 |
| #3 | 'complementary therap*' | 13060 |
| #2 | complementary AND therap* | 108091 |
| #1 | 'alternative medicine'/exp OR 'alternative medicine' | 98134 |

##### International HTA database

| Search query | Hits |
| --- | --- |
| ((acupuncture) OR (needling) OR (Complementary therapies) OR (Traditional Medicine)) OR ("Dry Needling"[mhe]) OR ("Acupuncture"[mhe]) OR ("Acupuncture Therapy"[mhe]) OR ("Complementary Therapies"[mhe]) | 199 |
| Filter: Chinese (Mandarin), Chinese (Cantonese), English | 139 |

##### CNKI

| Search query | Results |
| --- | --- |
| (SU="传统医药" OR SU="传统医学" OR SU="中医药" OR SU="中医技术" OR SU="针灸") AND (SU="卫生技术评估" OR SU="价值评估" OR SU="临床综合评价") | 68 |
| Translation from Chinese to English: (SU="Traditional medicine" OR SU=" Traditional thearies" OR SU="traditional Chinese medicine " OR SU="traditional Chinese medicine technology " OR SU="acupuncture") AND (SU="health technology assessment" OR SU="value assessment " OR SU="comprehensive clinical evaluation") | 68 |

##### Wanfang

| Search query | Results |
| --- | --- |
| 主题: (("传统医药" or "传统医学" or "中医药" or "中医技术" or "针灸") and ( "卫生技术评估" or "价值评估" or "临床综合评价" )) not 题名或关键词:("企业")  文献类型：期刊论文或学位论文 | 81 |
| Translation from Chinese to English: subject: (("Traditional medicine" or " Traditional therapies" or " traditional Chinese medicine " or " traditional Chinese medicine technology " or " acupuncture ") and ( " health technology assessment " or " value assessment " or " comprehensive clinical evaluation " )) not title or keywords:("Company")  literature type： journal paper or dissertation | 81 |
